# 

**Published:** 1896-12

**Authors:** 


### BOOKS RECEIVED.

The Practice of Medicine. A Text-Book for practitioners and students, with special reference to diagnosis and treatment. By James Tyson, M. D., Professor of Clinical Medicine in the University of Pennsylvania, and Physician to the Hospital of the University ; Physician to the Philadelphia Hospital; Fellow of the College of Physicians of Philadelphia, etc., etc. Octavo, pp. xvi.—1183. Illustrated. Price, $5.50. Philadelphia: P. Blakiston, Son & Company, 1012 Walnut street. 1896.

Medical Jurisprudence, Forensic Medicine and Toxicology. By R. A. Witthaus, A. M., M. D., and Tracy C. Becker, A. B., LL. B., and a staff of collaborators. In four royal octavo volumes. Volume IV., Toxicology. New York: William Wood & Company. 1896.

Twentieth Century Practice. An international encyclopedia of modern medical science. By leading authorities of Europe and America. Edited by Thomas L. Stedman, M. D., New York City. In twenty volumes. Volume VII., Diseases of the respiratory organs and blood, and functional sexual disorders. New York: William Wood & Company. 1896.

Anatomical Atlas of Obstetric Diagnosis and Treatment. By Oscar Schaeffer, M. D. Small octavo, pp. xii.—234. Illustrated. New York: William Wood & Company. 1896.

Essentials of Physical Diagnosis of the Thorax. By Arthur M. Corwin, A. M., M. D., Demonstrator of Physical Diagnosis in Rush Medical College, etc. Second edition, revised and enlarged. Philadelphia: W. B. Saunders, 925 Walnut street. 1896.

Twentieth Year-Book of the New York State Reformatory, for the fiscal year ending September 30, 1895. With illustrations and anthropometric tables. Elmira, N. Y. 1896.

Transactions of the Texas State Medical Association. Held at Fort Worth, Texas, April 28, 29, 30 and May 1, 1895. Printed by order of the association. 1896.

